# Three Cases of Croup, with Observations Tending to Point out the Contagious Nature of That Disease

**Published:** 1825-10

**Authors:** George Gregory

**Affiliations:** senior Physician of the St. George's and St. James's Dispensary.


**Art. VII.**—***Three Cases of Croup, with Observations tending to point out the Contagious Nature of that Disease.*** By George Gregory, M.D. senior Physician of the St. George's and St. James's Dispensary.

The sudden attack, the rapid advance, and the frequently fatal termination, of the complaint known by the name of croup, have long since attracted the attention of the profession, and given occasion to many Treatises, in which its history is ably laid down. None of those, however, to which I have had access, allude to any contagious property in the complaint, or inculcate the necessity, or even propriety, of separating a child affected by it from the healthy members of the family. It is with the view of drawing the attention of practitioners to this pathological fact, and its consequent practical inference, that I now lay the following cases before the readers of the Medical and Physical Journal.

On Thursday, December 4th, 1817, Mrs. Jackson, a respectable woman, residing in Portland-street, Soho, applied at the St. George and St. James's Dispensary, for advice for her eldest boy, Thomas Jackson, four and a half years old, whom she described as labouring under a severe attack of cynanche tonsillaris. On visiting the child, I found him extremely ill with ulcerated sore-throat, which had been present six days, and, by the mother's account, (which was, however, very indistinct,) had been preceded in its early stage by a scarlet eruption. The child had some cough and slight difficulty of breathing. The pulse was quick, and the bowels costive. Decoction of bark with acid, a purgative, and blister, were ordered.

Returning on the following day, I found that about midnight he had been seized with well-marked symptoms of croup. When I saw him, in the forenoon, the disease had evidently made great progress: the child was lying, its neck at the full stretch, gasping for breath; the face was pale and sunk, and the pulse exceedingly frequent. Leeches were applied to the throat, and calomel powders ordered to be given every two hours. The power of swallowing, however, soon failed, and the child died within twenty-four hours from the invasion of the croupy symptoms.

Permission being given to open the body, the trachea was found lined by a pretty thick tube of coagulating lymph, extending down nearly as far as the division of the bronchi. The larynx contained a considerable quantity of a dense glairy effusion. The trachea was removed, and submitted the same evening to the inspection of a number of gentlemen, who considered it as exhibiting a fine specimen of this remarkable morbid appearance.

I heard nothing more of the family until Wednesday, December 17th, when I was desired, late in the evening, to visit the second boy, John, ætatis three years, whom I had seen in my former attendance, and known to be a remarkably stout and healthy child. I was given to understand that the mother despaired of the child's life at the time she sent to me, desiring only to see me for her own satisfaction. I found him in the last stage of croup, the countenance pale and cadaverous, and the breathing sonorous and laborious. On inquiry, I ascertained that he had begun to complain on the Wednesday preceding,—that is, four days subsequent to the death of his brother; and that he had been attended, for several days past, by a medical practitioner in the neighbourhood. The croupy symptoms had been present nearly twenty-four hours. I did not think it advisable to do more than order the warm bath, which afforded considerable relief, and some calomel powders. A blister had been applied to the throat in the course of the morning. The child died in about nine hours; and, as the complaint was obviously the same with that of which the brother died, I did not think it requisite to ask for any examination of the body.

I now became very uneasy for the safety of the only remaining child, a remarkably fine girl, Ann, six years old, and I had some communication with the mother on the propriety of removing her from the room where these two unfortunate cases had occurred; but, as she must have been sent to a distance very inconvenient in the event of her being attacked by the same complaint, I advised that she should remain, and determined to watch her narrowly. In fact, on the following day, December 19th, symptoms of cynanche tonsillaris appeared; but the general health was not affected. On the 20th, the tonsils being more swelled, and some fever being present, I ordered six leeches to the throat, and directed an opening medicine and gargle. On the 21st, the tonsils were less swelled, deglutition was easier, and the breathing quite free. Early on the morning of the 24th, the breathing became sonorous, with great restlessness; and, when I saw her, which was towards one o'clock in the forenoon, in addition to these symptoms, vomiting had occurred, and the pulse was become exceedingly hard and wiry, and not less than 144 in the minute. With very great difficulty, owing to the restlessness and obstinacy of the little patient, the left external jugular vein was opened, and about eight ounces of blood taken away in a very quick stream. The child fainted, and became very sick at stomach; but the relief to the breathing was great and immediate. Three grains of calomel, with as many of antimonial powder, were ordered to be given every two hours. In the afternoon I again saw the child, in company with Dr. Holland, and found the croupy sound of the breathing returned; the pulse being again very hard, and excessively hurried. The heart was to be felt acting with great violence; the mouth and tongue parched. The same vein was again made to bleed, and a few ounces of blood abstracted, when vomiting came on. No buff appeared upon the blood in either case, but the coagulum was firm. In half an hour afterwards the warm bath was had recourse to, by which means the child passed a comfortable night. As the bowels were confined, an enema was ordered; and, to obviate a troublesome cough, a blister was applied to the chest.

On the 23d, the symptoms recurred with great violence, so that I found myself under the necessity (knowing the good effects of the former plan of treatment) of having again recourse to it. Six leeches were applied to the throat, the warm bath was repeated, and an opening mixture given. They had the desired effect; and in the evening the child was perfectly easy, and took some sago and wine. The calomel was discontinued.

She appeared better during the morning of the 24th, but the face was very pale, and the pulse exceedingly quick and sharp. Towards evening, restlessness came on, and the breathing became more laborious. I left her with directions that the throat should be fomented, and the warm bath repeated in the course of the night, if the symptoms were urgent.

On the 25th, the child was evidently sinking from exhaustion. I was given to understand that, during the night, the breathing became so exceedingly difficult, that (as a last resource) a few leeches were applied, the warm bath not having afforded any relief. Stimulants were now exhibited, which supported the child till about seven o'clock of that evening, when she died.

On dissection, a blush of inflammation appeared towards the upper rings of the trachea, with a considerable quantity of matter, of a purulent appearance, blocking up the passage; but no membrane, or any attempt at the formation of one, was to be observed. The lungs were in a perfectly healthy state.

I have to apologise for having been led into so long a detail of *treatment*, which to many must prove tedious, and to all, irrelevant to the professed object of the paper. The interest, however, with which I observed a young family cut off by this scourge of infant life, must plead my excuse.

It may remain a question, how far contagion had any share in the production of the disease in the two latter cases. The facts that I have brought forward must speak for themselves. I would only wish to add, that a family of nearly the same age, occupying the adjoining room, and which had been prevented, by the prudence of the mother, from having any communication with the affected children, has continued perfectly healthy; and I conclude the paper by expressing the firm determination of my own mind, on a full consideration of the circumstances of these cases, whatever may be the fate of the pathological opinion which I have advanced, to enforce most strictly, in every instance which may hereafter come under my care, the immediate separation of the affected infant from the healthy children of the family.

*Upper John-street; September* 10.

